# Content Analysis of Hospitals’ Community Health Needs Assessments in the Most Violent Cities: 2023 Update

**DOI:** 10.5811/westjem.48501

**Published:** 2025-07-18

**Authors:** Ai Alexa Tarui, Robert D. Flint, Benoit Stryckman, William Wical, Henry D.M. Schwimmer, Kyle Fischer

**Affiliations:** * University of Maryland School of Medicine, Department of Emergency Medicine, Baltimore, Maryland; † Johns Hopkins Bloomberg School of Public Health, Department of Health Policy and Management, Center for Gun Violence Solutions, Baltimore, Maryland; ‡ Alameda Health System, Department of Emergency Medicine, Oakland, California

## Introduction

The Patient Protection and Affordable Care Act created a mandate for non-profit hospitals to conduct community health needs assessment (CHNAs) every three years. These allow researchers to conduct empirical analyses of hospitals’ efforts to address issues of violence prevention. This study performs an analysis of CHNAs from hospitals within the twenty most violent U.S. cities and compares the content of CHNAs released after the ACA was implemented to hospitals’ most recent CHNAs. Eight-seven CHNAs were analyzed for specific violence-related keywords and the designation of violence as an overall health need.

## Methods

The selection criteria of hospitals and identification of trauma center status was previously described in Fischer et al.^1^ The same cohort of hospitals were analyzed with their most recent CHNA from 2019–2023. CHNAs were collected between May and June of 2023. We investigated changes over time. This allows an analysis of temporal trends in the hospital identification of violence as a health issue.

To standardize the coding done by different individuals, we all coded for three of the same CHNAs and compared our results, which were the same. Each individual CHNA was coded based on the inclusion of the terms “violence”, “violent”, “assault”, “murder”, “homicide”, or “intentional injury”. Each was further characterized by the type of violence --community, domestic or sexual, child abuse, or terrorism. Moreover, statistics on the burden of violent injuries were reported, along with potential causes of the violence or if violence was recognized as a priority need by external stakeholders like non-hospital personnel or community members. Each CHNA was assessed for the presence or absence of violence listed as an overall community health need. We used a two-tailed Fisher’s exact test to compare CHNAs from hospitals with and without trauma centers. Our primary outcome measure was to examine whether the same hospitals (trauma vs non-trauma centers) in the twenty most violent US cities have identified violence prevention as a major health need in their most updated CHNAs collected in 2023. Our secondary outcome was to compare in the specific ways the hospitals include violence-related terminology, the types of violence referenced, and how often external stakeholders raise the issue between the CHNAs gathered in 2015 vs 2023.

## Results

87 hospitals were identified and 19 of these hospital CHNAs did not meet the inclusion criteria, leaving a sample size of 68 hospital CHNAs.

Of the 68 hospitals, 58 (85%) CHNAs had violence-related terms mentioned with the most widely used terms used being “violence” 54 (93%) and “violent” 38 (66%). Forty-five (66%) CHNAs provided statistics about the burden of violence and 23 (34%) described potential causes of violence. External stakeholders identified violence as a community health issue in 43 (63%) CHNAs. Overall, violence was identified as a health need in 38 (56%) hospitals (Table 1).

When comparing the results from CHNAs collected from 2019–2023 to those from 2010–2016, violence-related terms were mentioned at a higher rate in the new data set, with 58 (85%) CHNAs mentioning them in the new paper versus 57 (74%) in the prior (Table 2). Of the terms used, only violence in reference to intimate partner/domestic/sexual violence (76% vs 42%) increased. Statistics about the burden of violence (66% vs 56%) increased. Additionally, violence identified as a community health need by external stakeholders (63% vs 38%) increased. Overall, the new data set also had an increase in violence being identified as a health need (56% vs 32%) (Table 3). A limitation is that the presence of a term does not indicate the intensity to which violence was addressed nor does the absence exclude the possibility that hospitals are not participating in community outreach work.

## Conclusions

These results demonstrated a significant increase from prior CHNAs for the identification of violence as a health need (56% vs 32%) and usage of violence-related terms (85% vs 74%). This study suggests that although there has been an increasing recognition of violence as a health issue, there is a need for additional education and programming for community responses to violence.

This represents an opportunity to improve the care for violently injured patients as well as programming. Overall, public health professionals must educate the public, health professionals, and policymakers on the importance of health-based strategies for violence prevention.

## Figures and Tables

**Table 1:** Results of CHNAs collected 2019–2023.

Content present in CHNAs	All (N=68)	Trauma Centers (N=46)	Non-trauma center (N=22)	p-value
Violence-related terminology utilized	85.3% (58)	87.0% (40)	81.8% (18)	0.72
Statistics on burden of violence provided	66.2% (45)	70.0% (32)	59.1% (13)	0.42
Underlying cause of violence discussed	33.8% (23)	32.6% (15)	36.4% (8)	0.79
External stakeholders perceive of violence as a need	63.2% (43)	65.2% (30)	59.1% (13)	0.79
Violence is designated as an overall priority need	55.9% (38)	60.1% (28)	45.5% (10)	0.30

*CHNAs*, community health needs assessment.

**Table 2:** Violence related terms in CHNAs collected in 2010–2016 v 2019–2023.

	Violence related terms	“Violence”	“Violent”	“Homicide”	“Murder”	“Assault”	“Intentional injury”
2010–2016 CHNAs	74.0% (57/77)	73.7% (42/57)	43.9% (25/27)	61.4% (35/57)	19.3% (11/57)	24.6% (14/57)	0% (0/57)
2019–2023 CHNAs	85.3% (58/68)	93.1% (54/58)	65.5% (38/58)	48.3% (28/58)	19.0% (11/58)	48.3% (28/58)	19.0% (11/58)

*CHNAs*, community health needs assessment.

**Table 3:** Results of CHNA collected in 2010–2016 vs 2019–2023.

	Violence in reference to community violence	Violence in reference to dating/domestic/intimate partner or sexual violence	Violence in reference to child abuse	Statistics on burden of violence provided	Underlying causes of violence discussed	External stakeholders’ perception of violence as a need	Violence is designated as an overall priority need
2010–2016 CHNAs	87.7% (50/57)	42.1% (24/57)	22.8% (13/57)	55.8% (43/77)	16.9% (13/77)	37.7% (29/77)	32.5% (25/77)
2019–2023 CHNAs	86.2% (50/58)	75.9% (44/58)	6.9% (4/58)	66.2% (45/68)	33.8% (23/68)	63.2% (43/68)	55.9% (38/68)

*CHNAs*, community health needs assessment.
